# Is Curcumin the Answer to Future Chemotherapy Cocktail?

**DOI:** 10.3390/molecules26144329

**Published:** 2021-07-17

**Authors:** Wei-Yang Kong, Siew Ching Ngai, Bey-Hing Goh, Learn-Han Lee, Thet-Thet Htar, Lay-Hong Chuah

**Affiliations:** 1 School of Biosciences, Faculty of Science and Engineering, University of Nottingham Malaysia, Semenyih 43500, Selangor, Malaysia; leonkong93@gmail.com (W.-Y.K.); eunice.ngai@nottingham.edu.my (S.C.N.); 2 Biofunctional Molecule Exploratory Research Group, School of Pharmacy, Monash University Malaysia, Bandar Sunway 47500, Selangor, Malaysia; goh.bey.hing@monash.edu (B.-H.G.); thet.thet.htar@monash.edu (T.-T.H.); 3 College of Pharmaceutical Sciences, Zhejiang University, 866 Yuhangtang Road, Hangzhou 310058, China; 4 Novel Bacteria and Drug Discovery (NBDD) Research Group, Microbiome and Bioresource Research Strength, Jeffrey Cheah School of Medicine and Health Sciences, Monash University Malaysia, Bandar Sunway 47500, Selangor, Malaysia; lee.learn.han@monash.edu

**Keywords:** cancer, curcumin, combination therapy, chemotherapy cocktail, signalling pathways, anticancer

## Abstract

The rise in cancer cases in recent years is an alarming situation worldwide. Despite the tremendous research and invention of new cancer therapies, the clinical outcomes are not always reassuring. Cancer cells could develop several evasive mechanisms for their survivability and render therapeutic failure. The continuous use of conventional cancer therapies leads to chemoresistance, and a higher dose of treatment results in even greater toxicities among cancer patients. Therefore, the search for an alternative treatment modality is crucial to break this viscous cycle. This paper explores the suitability of curcumin combination treatment with other cancer therapies to curb cancer growth. We provide a critical insight to the mechanisms of action of curcumin, its role in combination therapy in various cancers, along with the molecular targets involved. Curcumin combination treatments were found to enhance anticancer effects, mediated by the multitargeting of several signalling pathways by curcumin and the co-administered cancer therapies. The preclinical and clinical evidence in curcumin combination therapy is critically analysed, and the future research direction of curcumin combination therapy is discussed.

## 1. Introduction

Cancer has remained a serious threat to public health worldwide for centuries, and its prevalence is multiplying at a worrying rate. According to global cancer statistics (GLOBOCAN) 2020, there will be about a 47% increase in cancer incidence in 2040 as compared to the estimated cases in 2020. The growing burden of cancer cases will likely correspond to the mortality rate among cancer patients, especially breast cancer and colorectal cancer [1]. With continuous collaborative research efforts between scientists and clinicians, multiple treatment modalities have been developed and improved throughout the years to control cancer aggravation, for instance, surgery, chemotherapy, radiotherapy and immunotherapy [2]. Fortunately, these treatment interventions have led to a decline in the mortality rate for cancer patients. However, not all cancer patients benefit completely from these treatments, owing to the difference in susceptibility to cancer treatment, heterogeneity of cancer, therapeutic resistance development and unprecedented side effects [3]. Hence, more initiatives are needed in the quest for more potent treatment modalities for a wide range of cancer patients to overcome the therapeutic obstacles.

Over the years, the concept of combination therapy, which relies on two or more therapeutic agents, has been introduced in the development of cancer treatment [4]. Compared to conventional single-agent therapies, combination therapy exhibits numerous benefits, such as targeting multiple oncogenic pathways, reducing high toxicity levels implicated by monotherapy, improving the magnitude of therapeutic responses and reducing the likelihood of therapeutic resistance [5]. These benefits allow for the greater suppression of cancer cells and reduce the risk of cancer recurrence, thus improving the patient’s quality of life [4,6]. Lately, curcumin has gained a great deal of interest, attributed to its broad range of medicinal properties [7]. Intriguingly, curcumin exhibited countless anticancer properties, such as limiting cancer cell proliferation, promoting tumor cell death and preventing metastasis [7,8]. Besides, curcumin supplementation greatly relieves the patients from experiencing adverse effects caused by conventional therapies [6]. Hence, these properties pose great advantages to the development of curcumin combination therapy for cancer treatment.

This review focuses on the use of curcumin in combination therapy in various cancers. The evasive mechanisms developed by cancer cells in response to cancer therapy are discussed. Curcumin combination therapies used are reviewed in depth in each type of cancer in both preclinical and clinical studies. We also addressed how curcumin modulates a variety of molecular targets in cancer cells in the combination treatment, to provide an insight into the multitargeting effects of such treatment cocktails.

## 2. Evasive Mechanisms and Chemoresistance

**Figure 1:**
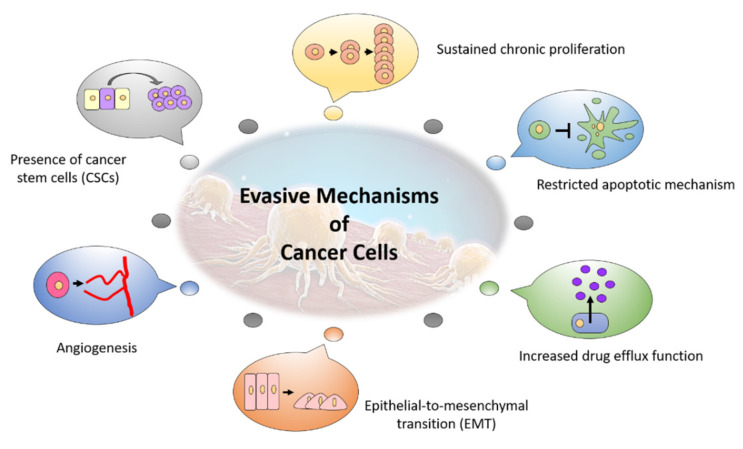
The evasive mechanisms developed by cancer cells in response to cancer therapy.

Though the development of cancer therapy has achieved progressive milestones from conventional chemotherapeutic agents to the more advanced monoclonal antibodies and immunotherapy, inconsistencies and irresponsiveness towards the therapies were observed among the cancer patients receiving various cancer treatments, resulting in mixed outcomes among patients. Owing to the neoplastic characteristics, cancer cells are continuously evolving and adapting to the stress response induced by cancer therapies, developing numerous evasive mechanisms pertaining to the hallmark of cancer as their defensive strategies against chemotherapy [9]. Figure 1 summarizes the different evasive mechanisms by cancer cells.

### 2.1. Sustained Chronic Proliferation

Cancer is notorious for the sustained chronic proliferation behaviour in the body [9]. It is capable of neutralizing the cytotoxicity effects by profoundly accelerating cell proliferation to promote survival benefits. This uncontrolled cell growth is greatly manifested by the constitutive activation of several proliferation-related signalling pathways, notably transforming growth factor-β (TGF-β), phosphoinositide 3-kinase(PI3K)/protein kinase B(Akt)/mechanistic target of rapamycin (mTOR), epidermal growth factor receptor (EGFR), mitogen-activated protein kinase (MAPK) and nuclear factor κ-light-chain-enhancer of activated B cells (NF-κB) pathways in cancer cells [3,10]. Additionally, cancer cells induce aberrant cell growth through the dysregulation of the cell cycle and modulation of cell cycle-related proteins. Evading the cancer therapy, cancer cells usually instigate high activities of cyclin-dependent kinase (CDK), with the abnormal amplification of cyclin D, to accelerate the cancer cell proliferation [11,12]. Concurrently, the uncontrolled cell cycle progression is tightly associated with the decreased expression or loss of function of CDK inhibitors such as p21 and p27 [13]. Apart from its intrinsic mutation in cancer cells, tumor suppressor protein p53 is frequently downregulated in response to cancer therapy, thereby abolishing its anti-proliferative, anti-apoptosis and anti-metastatic activities [14].

### 2.2. Restricted Apoptotic Mechanism

Restricting the apoptotic mechanism is another strategy of cancer cells to override the killing effects induced by conventional therapies. As an orchestrated event in regulating physiological function, apoptosis mainly involves the extrinsic death receptor (DR) pathway and intrinsic mitochondrial pathway, which are tightly regulated by the balance of apoptotic proteins [15]. To maintain its immortality and metastatic behaviours, cancer cells have established several mechanisms to avoid apoptosis induction by cancer therapies [16]. As extensively reviewed in the literature, the fate of cell death is modulated by the balance of B-cell lymphoma 2 (Bcl-2) family proteins, which are composed of pro-apoptotic proteins, such as Bcl-2 homology 3 (BH3)-interacting domain death agonist (Bid), BH3-only Bcl-2-interacting mediator of cell death (Bim), Bcl-2-associated X protein (Bax), Bcl-2 homologous antagonist/killer (Bak) and Bcl-2-interacting killer (Bik), and anti-apoptotic proteins, such as Bcl-2, B-cell lymphoma-extra-large (Bcl-xL), induced myeloid leukemia cell differentiation protein (Mcl-1), and Bcl-2-like protein 10 (Bcl2L10) [17]. The ratio of Bax/Bcl-2 is sometimes applied as the prognostic marker and susceptibility of a particular cancer treatment to cancer patients [18,19]. The overexpression of Bcl-2 is highly correlated with poor therapeutic efficacy among cancer patients [20]. Besides, the inhibitor of apoptosis (IAP) protein families, including cellular inhibitor of apoptosis protein 1 (cIAP1), cellular inhibitor of apoptosis protein 2 (cIAP2), X-linked inhibitor of apoptosis protein (XIAP) and survivin, are important determinants in the restriction of cancer cell death. These IAPs bind to the active site of caspases, thus limiting caspase activation and apoptosis induction [16]. Furthermore, the impairment of the extrinsic DR pathway was demonstrated by cancer cells to evade the apoptosis induced by chemotherapies. Apart from the downregulation of DRs, the reduced surface expression of DRs due to endocytosis was initiated by cancer cells, limiting the sensitivity of cancer cells towards drug treatments [21,22].

### 2.3. Increased Drug Efflux Function

The drug efflux mechanism is increased in cancer cells as a means to escape drug cytotoxicity effects [23]. The drug efflux function is governed by multidrug resistance protein (MDR), which belongs to the ATP binding cassette (ABC) transport families, notably P-glycoprotein (P-gp) (also known as multidrug resistance protein 1 (MDR1)), multidrug resistance-associated protein 1 (MRP1) and breast cancer resistance protein (BCRP) [24]. The overexpression of MDRs is found in a considerable number of cancers with both intrinsic and acquired resistance [25]. Furthermore, the upregulated expression of oncogenic kinases, such as MAPK and extracellular-signal-regulated kinase (ERK) signalling, caused the elevation of MDR expression in cancer cells, thus limiting the drug sensitivity. Apart from preventing the accumulation of chemotherapeutic drugs, these MDRs influence the pharmacokinetics, such as drug biodistribution, drug metabolism and toxicity, on the drug treatment [26,27]. Albeit many MDR-targeting therapeutics were developed to circumvent the chemoresistance issue, but the clinical benefits were not well justified due to high toxicity and underreporting of MDR protein inhibition in patient samples [24].

### 2.4. Epithelial to Mesenchymal Transition

Characterized by the dissolution of adherens junctions and loss of cell–cell contacts, the Epithelial to mesenchymal transition (EMT) process is tightly modulated by TGF-β/Smad signalling, which directs the downstream regulation of Snail, zinc finger E-box-binding homeobox (ZEB) and basic helix–loop–helix (bHLH) transcription factor families [28,29]. Concomitantly, this causes the loss of epithelial markers E-cadherin, while acquiring the mesenchymal features through upregulation of N-cadherin and vimentin [30]. In addition to that, EMT potentiates the migration and invasive behaviours of cancer cells to other parts of tissues. The expression level of the aforementioned biomarkers is important in determining the sensitivity of chemotherapeutic drug treatments in cancer cells [9,28]. The elevation of E-cadherin expression restored the sensitivity to tyrosine kinase inhibitors (TKIs) in lung cancer stem cells (CSCs) [31]. Furthermore, it has been shown that patients with mesenchymal-like urothelial cancer cells had the worst prognosis as compared to patients with epithelial luminal tumors [32]. These studies suggest that the emergence of EMT in cancer cells are prone to defence against chemotherapeutic drugs.

### 2.5. Angiogenesis

Angiogenesis also plays a major role in the chemoresistance capability of cancer cells. During metastasis, angiogenesis was initiated to sustain its nutrients and oxygen supplies for cancer cells [9,28]. This phenomenon is modulated by an unbalanced mix of multifaceted angiogenic regulators, including the aberrant amplification of vascular endothelial growth factor (VEGF) and the dysregulation of matrix metalloproteinase (MMP) activities, thus enabling the matrix remodelling, metastasis of cancer cells to other tissues, and microvessel formation [33]. Furthermore, in order to survive in an oxygen-deprived condition, cancer cells had augmented the expression of hypoxia-inducible factor 1-α (HIF-1α) which could, in turn, stimulate pro-angiogenic activities [34]. The upregulation of pro-angiogenic activities has promoted therapeutic resistance by hindering cell death, preparing for a pro-survival mechanism and directing metastasis in tumor cells [35].

### 2.6. Cancer Stem Cells (CSCs)

Being self-renewable with differentiation potency, CSCs have been recognized as the crucial factor in rendering cancer treatment failure [36]. Despite the bulk elimination of tumor cells by chemotherapy, this small population of CSCs within the tumor cell pool exhibits their unlimited proliferative and survival capacities to establish more powerful metastatic and invasive characteristics, causing cancer to relapse [3]. Furthermore, CSCs survive the stress of chemotherapy by developing several resistance mechanisms, notably the inhibition of apoptosis, protection against oxidative DNA damage and induction of EMT [37]. Drug accumulation in CSCs was also countered by the enhanced expression of MDR, thereby limiting the drug cytotoxicity effects [23,38]. Besides, CSCs cause the massive upregulation of numerous oncogenic signalling pathways, such as Wnt/β-catenin, Notch, PI3K/Akt/mTOR signalling pathways, in order to sustain its proliferative, metastatic and chemoresistance potential, thereby overriding the chemotherapeutic drug effects [36]. As CSCs reside within a tumor niche, the interaction with cancer-associated fibroblasts and tumor-associated macrophages promotes the secretion of numerous cytokines, transcription factors and growth factors within the tumor microenvironment, triggering CSCs to become more resistant to cancer treatment [39].

## 3. Curcumin and Its Medicinal Properties

Curcumin, also regarded as diferuloylmethane, is a yellow polyphenol extracted from the rhizome of the Curcuma longa (turmeric) plant, belonging to the Zingiberaceae family [40]. Indigenous in south-eastern and southern tropical Asia, curcumin is vastly utilized for food preservation, colouring and flavouring in daily activities [41]. Moreover, curcumin is traditionally applied for pain-relieving and wound healing effects. Commercial curcumin products contain approximately 77 % curcumin, 18 % demethoxycurcumin and 5 % bisdemethoxycurcumin [42]. Out of those curcuminoids, curcumin exhibits the most potent medicinal properties as compared to demethoxycurcumin and bisdemethoxycurcumin [41]. A growing body of evidence has demonstrated the benefits of curcumin in treating various diseases, including metabolic syndromes, hyperlipidaemia, inflammatory skin conditions, neurodegeneration and rheumatoid arthritis. These clinical benefits are attributed to the anti-inflammatory, anti-oxidant, and wound healing activities of curcumin [2,8,40]. Moreover, curcumin can impede pathogenic infections by exerting a broad spectrum of anti-bacterial, anti-fungal and anti-viral activities [43]. Alongside profound medicinal properties, curcumin is listed as a “generally recognized as safe (GRAS)” compound by the Food and Drug Administration (FDA), supporting its safety and tolerability when consumed by patients [7].

**Figure 2:**
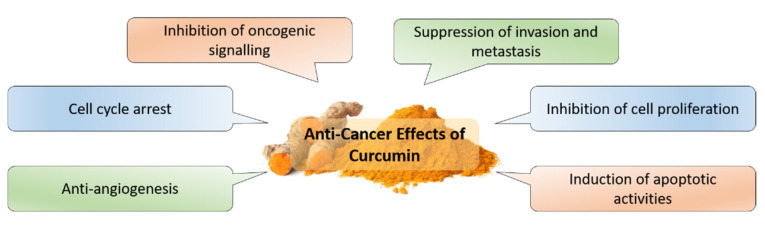
Curcumin elicits various anticancer effects in cancer treatment.

Enormous attention has given to the exploration of anticancer properties in curcumin. To date, curcumin has shown its anticancer benefits in numerous cancers such as breast cancer [44,45], colorectal cancer [46], lung cancer [47], pancreatic cancer [48] and prostate cancer [49]. In fact, these anticancer effects depicted by curcumin are highly associated with the modulation of several oncogenic signalling pathways, which are essential in cancer development. Curcumin constrains these oncogenic signalling pathways and further limits the downstream pro-tumorigenic activities. In vitro studies illustrated that curcumin treatment limited the proliferation and caused cell cycle arrest in HT-29 colon cancer cells and PLC/PRF/5 liver cancer cells via the inhibition of cyclin D1, with the downregulation of NF-κB and cyclooxygenase-2 (COX-2) signalling [50,51,52,53]. Concurrent with the upregulation of tumor suppressor gene p53, curcumin repressed the proliferative potential of cancer cells via the downregulation of PI3K/Akt/mTOR signalling [54,55,56]. Furthermore, it also impedes cancer cells’ survival and suppresses their metastatic ability through the downregulation of EGFR pathways [57,58] and inhibition of MMP activities [59,60]. Apart from limiting the expression of IAP family proteins, curcumin promotes the apoptosis of cancer cells by increasing the expression of Bax while downregulating the expression of Bcl-2 in various cancer cells [61,62]. It has also been shown to be able to abrogate angiogenesis elicited by breast tumors via the suppression of VEGF [63]. The anticancer properties elucidated by curcumin are summarized in Figure 2.

## 4. Curcumin Combination Anticancer Therapy in Preclinical Studies

Conventional cancer therapies are challenged by the various defence mechanisms developed in cancer cells, hindering treatment success. Moreover, cancer cells often exhibit more than one mechanism of resistance, further complicating the treatment regimen. Hence, multiple targeting of these evasive mechanisms could potentially restore the sensitivity of cancer cells to cancer therapy, apart from eliminating the cancer cells completely. In this regard, curcumin holds a great promise in combination therapy to enhance the anticancer effects, while circumventing the problems encountered in conventional therapies. Since curcumin exhibits pleiotropic effects, the co-administration of curcumin in cancer therapy allows multiple targeting to the cancer-surviving and cancer-limiting mechanisms, while conventional monotherapy is restricted by single mechanism targeting only. Moreover, repetitive monotherapy caused the cancer cells to recruit other salvage pathways for survival benefits [4]. Hence, curcumin combination therapy could offset the evasion of cancer therapy and survivability of cancer cells, thereby overcoming the risk of cancer recurrence and treatment failure. To ensure the complete elimination of cancer cells, conventional monotherapy is often administered at a high dosage and causes a series of unwanted side effects [5]. Moreover, it was well known to be non-selective in killing proliferating cancer cells as well as healthy normal cells, upsetting the body immune system, and resulting in high toxicity [4]. These drawbacks could be alleviated by co-administering curcumin, such that a lower dosage of therapeutic agents is required. Thus, this could reduce the toxicity and adverse effects encountered by patients, besides yielding a significant therapeutic response [6,64]. To date, numerous preclinical investigations involving in vitro, in vivo and ex vivo studies have been conducted on the use of curcumin in combination therapy in various cancers (Table 1).

### 4.1. Curcumin Combination Therapy in Breast Cancer

Compelling evidence has demonstrated the benefits of curcumin combination therapy as compared to monotherapy in breast cancer. As a selective estrogen receptor modulator, tamoxifen is renowned for the treatment of hormone-positive breast cancer [144]. Nonetheless, repeated treatments confer chemoresistance, attributed to the dysregulation of cell cycle and interruption on multiple signal transduction pathways [145]. An in vitro investigation reveals that the co-administration of curcumin and 4-hydroxytamoxifen (4-OHT), a metabolite of tamoxifen, could restore the sensitivity of 4-OHT of HR-positive MCF-7 cells through the downregulation of cyclin D1 and upregulation of p21. Concomitantly, the cell proliferative effect was reduced significantly via the repression of Akt/mTOR signalling pathways. Compared to either curcumin or 4-OHT alone, combined treatment also remarkably activated pro-apoptotic protein Bcl-xL and suppressed the Bcl-2 proteins, thereby further enhancing the apoptotic activities [65].

Apart from that, the Snail-related zinc-finger transcription factor (SLUG) overexpression, which is correlated to poor prognosis in various cancers [146,147], has been linked to tamoxifen resistance in breast cancer therapy [146]. The phenomenon was reversed with the combined treatment of curcumin and 4-OHT in MDA-MB-231 cells. Besides weakening mTOR activities, the reversal of chemoresistance was accompanied by enhanced mitochondrial-mediated apoptosis and the downregulation of hexokinase 2 (HK2) activities, therefore mediating cell death and preventing the metastatic behaviour of breast cancer cells, respectively [66].

Human epidermal growth factor receptor-2 (HER2) overexpression accounts for 15–30% of metastatic breast cancer, which exacerbates aberrant cell proliferation and cell survival in breast cancer patients [148]. To date, trastuzumab, an anti-HER2 monoclonal antibody, serves as the most efficacious targeted therapy in treating HER2-related breast cancer [149]. It was shown to be able to maximize its therapeutic potential when combined with other anticancer agents [150,151]. Through cell proliferation and cell cycle analysis, co-treatment of curcumin (10 μg/mL) and trastuzumab (10 μg/mL) significantly reduced cell proliferation and induced G2/M arrest in HER2-overexpressed BT-474 and SK-BR-3-hr (a herceptin resistant strain from SK-BR-3) breast cancer cells, compared to trastuzumab alone. This was accompanied by the suppression of HER2 expression with the inhibition of downstream targets such as NF-κB, Akt and MAPK signalling pathways. Further in vivo study revealed that BT-474 xenograft mice models had the smallest tumor volume after 4 weeks of curcumin (45 mg/kg) and trastuzumab (4 mg/kg) co-treatment, when compared to curcumin or trastuzumab alone [67].

Curcumin also serves as a potential adjuvant with other chemotherapeutic agents in augmenting anticancer effects. The combined treatment of curcumin and paclitaxel significantly suppressed the paclitaxel-mediated NF-κB expression and its regulatory genes COX-2, matrix metallopeptidase 9 (MMP-9), VEGF, and intercellular adhesion molecule 1 (ICAM-1), thus promoting the anti-proliferative and anti-metastatic behaviour in breast cancer cells [68,152]. Interestingly, further experiments proved that curcumin and paclitaxel curbed the metastasis of MDA-MB-435 breast cancer cells to lung tissues in xenograft mice models [69]. More importantly, this combination of curcumin (ranging from 25–225 mg/kg) and paclitaxel (5 mg/kg) was found to be safe and induced no toxicity effects in mice models [68].

Hindered by drug efflux and chemoresistance, doxorubicin was explored in combination with curcumin in breast cancer treatment [26]. A study reported that this co-treatment profoundly blocked the drug efflux function, as influenced by ATP binding cassette subfamily B member 4 (ABCB4) [72]. Additionally, the co-delivery of doxorubicin with curcumin loaded in solid lipid nanoparticles was shown to inhibit the tumor growth in mice by decreasing the P-gp surface expression besides increasing intracellular retention of doxorubicin. This has successfully augmented the cytotoxicity effect on breast cancer cells [73]. Besides, another study illustrated that curcumin inhibited the doxorubicin-induced EMT via the suppression of Akt, β-catenin and glycogen synthase kinase 3 β (GSK3β) protein expression, emphasizing the importance of the combined treatment of curcumin and doxorubicin in inhibiting the metastasis of breast cancer cells [153].

Apart from the combination with chemotherapeutic agents, the combined treatment of curcumin with other natural compounds has also been investigated in breast cancer. Flow cytometry cell death analysis showed that the co-treatment of curcumin (5 μM) and berberine (25 μM) synergistically exerted apoptosis and autophagy cell death to MDA-MB-231 and MCF7 breast cancer cells [76]. Moreover, curcumin (1.5 μM) sensitized the AU565 breast cancer cells treated with quercetin (4 μM) and optiberry (2 μg/mL) to decrease lapatinib-mediated HER2 overexpression via the downregulation of HER2/Akt signalling pathways [74]. Another study reported the benefits of curcumin (200 mg/kg) and epigallocatechin gallate (EGCG) (25 mg/kg) in lowering the tumour burden of xenograft models via the reduction in phosphorylated Akt, EGFR and vascular endothelial growth factor receptor-1 (VEGFR-1) expression, highlighting the enhanced anticancer potential of this treatment regimen [75].

### 4.2. Curcumin Combination Therapy in Colorectal Cancer

To date, 5-fluorouracil (5-FU) remains one of the first-line treatments for colorectal cancer patients. Unfortunately, its clinical efficacy was limited by the low (about 10-15%) overall response in metastatic colorectal cancer patients [154]. Moreover, the toxicity experienced with increasing dosage of 5-FU has caused further constraints on the treatment [155]. Growing evidence has proven the role of curcumin in benefiting the chemotherapeutic efficacy of colorectal cancer preclinically. For instance, the toxicity profile was alleviated when the co-treatment of 5 μM curcumin lowered the concentration of 5-FU (0.01 nM; originally 10 nM) required in diminishing the cell proliferation of 3D cell culture models [77]. Concurrently, this was associated with G0/G1 cell cycle arrest via cyclin D1 inactivation [78,79]. Another cell cycle study showed that the co-treatment of curcumin and 5-FU caused S phase arrest in 5-FU-resistant HCT116 cell lines, implying that the regulation of cell cycle is cell-specific [80]. Furthermore, invasion assays revealed that this treatment combination reduced the migratory behaviour of colorectal cancer cells via the inhibition of MMP-9 and C-X-C chemokine receptor type 4 (CXCR4) expression, the downregulation of NF-κB expression, and disruption of ten–eleven translocation methylcytosine dioxygenase 1 (TET1)–naked cuticle homolog 2 (NKD2)–Wnt signalling pathways [77,79]. Apart from that, they also promoted the apoptotic activities in colon cancer cells via the upregulation of Bax and the cleavage of caspase 3, 8 and 9 [78,80]. Another study proved that 5-FU-induced autophagy was inhibited in HCT116 and HT29 colon cancer cells co-treated with curcumin and 5-FU, via the downregulation of Akt and mTOR activities [81]. The progression of 5-FU therapeutic resistance, either intrinsically or by repeated treatments, had exacerbated the uncontrolled cell division and survival of colorectal cancer cells via the elevation of pro-oncogenic NF-κB, Wnt and PI3K/proto-oncogene tyrosine–protein kinase (Src) signalling. However, this phenomenon was resolved when the colorectal cancer cells were co-treated with curcumin and 5-FU [77,79,80].

Other FDA-approved standard chemotherapeutic agents for colorectal cancer treatment include oxaliplatin, dasatinib and irinotecan [155]. Several preclinical studies demonstrated the improvement in therapeutic efficacy of colorectal cancer cells when these chemotherapeutic agents are co-administered with curcumin. In addition to increased growth inhibitory effects, the combined treatment of curcumin and other chemotherapeutic drugs, such as oxaliplatin or irinotecan, significantly promoted apoptotic activities in HT29 and HCT116 colon cancer cells via the increased reactive oxygen species (ROS) production and the upregulation of endoplasmic reticulum-associated protein C/EBP homologous protein (CHOP) [82,83]. Besides, the suppression of TGF-β, Smad-2 and N-cadherin indicated the EMT abolishment in colorectal cancer cells co-treated with curcumin and oxaliplatin [82,84,85]. The presence of CSCs in tumor microenvironment supports the notion of therapeutic failure and resistance. Therefore, targeting the CSCs can prevent cancer recurrence followed by tumor eradication [156]. The repeated administration of dasatinib and irinotecan has been shown to cause chemotherapeutic resistance in colorectal cancer cells. The co-administration of these drugs with curcumin had inhibited the sphere-forming potential of colorectal CSC through the downregulation of CSC markers, such as CD44, CD133, epithelial cell adhesion molecule (EpCAM) and CD24, indicating that curcumin can alleviate the cancer progression by interrupting with CSCs activities when co-treated with other chemotherapy agents [84,85].

The combined treatment of multiple conventional chemotherapeutic agents has produced positive clinical outcomes in colorectal cancer patients to some extent. However, despite the improved overall survival and response rate initially, gradual irresponsiveness to repetitive treatment and modest toxicity were also experienced by the patients [155]. The combination of 5-FU and oxaliplatin were shown to elevate the expression of tumour-promoting proteins, such as EGFR, HER2, human epidermal growth factor receptor 3 (HER3), Akt and COX2, which rendered the cell survival and uncontrolled cell proliferation in chemo-surviving cells. Nevertheless, these tumor-promoting effects were curbed by the co-administration of curcumin [86,87]. Interestingly, the supplementation of curcumin increased the expression of insulin-like growth factor-binding protein 3 (IGFBP3) and promoted the binding between IGFBP3 and insulin-like growth factor 1 (IGF-1), sequestering the activation of IGF-1 and inhibiting the IGF-1/insulin-like growth factor type 1 receptor (IGF-1R) signalling, in 5-FU and oxaliplatin-treated colorectal cancer cells [86,87,94,95]. Furthermore, ex vivo spheroid cultures showed a great reduction in cell proliferation and the decreased expression of stem cell markers, such as Nanog, vascular endothelial growth factor receptor-2 (VEGFR-2) and octamer-binding transcription factor 4 (Oct4), with the combined treatment of 5-FU and oxaliplatin with curcumin. Further clinical studies have demonstrated that curcumin supplementation of 0.5–2 g/day to 5-FU- and oxaliplaitin-treated patients were clinically safe [88]. Moreover, the co-administration of curcumin and 5-FU and oxaliplatin significantly suppressed EGFR signalling via the increased methylation status of EGFR, emphasizing the role of curcumin in the epigenetic modulation of colon cancer cells [89]. On the other hand, the combination of bevacizumab (0.4 mg/kg) and turmeric extract (with absorbable curcumin) (400 mg/kg) caused an inhibition of angiogenesis while promoting apoptosis in the tumor area in the xenograft model. Besides, the absence of systemic toxicity to the liver, kidney and heart in in vivo models signifies the safety and tolerability of this combined therapy [90].

Montgomery et al. (2016) reported that curcumin potentiated the anticancer effects of other natural compounds, where curcumin (12.5 μM) and silymarin (12.5 μM) synergistically augmented the apoptosis of DLD-1, HCT116 and LoVo colon cancer cells with increased caspase 3/7 activities, as compared to monotherapy [91]. Another study involving (3-(4,5-dimethylthiazol-2-yl)-2,5-diphenyltetrazolium bromide) tetrazolium (MTT) assay and cell cycle analysis revealed that the co-treatment of curcumin (10 μM) and resveratrol (10 μM) showed synergistic anticancer effects in HCT116 colon cancer cells by inhibiting cell proliferation and inducing S phase arrest. This was associated with the reduction in oncogenic protein expression, notably EGFR, HER2, HER3 and IGF-1R levels. Further in vivo studies confirmed tumor growth reduction without any systemic toxicity, indicating the safety of curcumin and resveratrol [92].

### 4.3. Curcumin Combination Therapy in Lung Cancer

Current standard chemotherapeutic agents for non-small-cell lung carcinoma (NSCLC) patients are TKIs, such as gefitinib and erlotinib. Chemoresistance towards this treatment is becoming more prevalent, where the abnormal amplification of EGFR was massively identified in NSCLC patients [157]. Flow cytometric apoptotic analysis revealed that the co-treatment of curcumin (10 ng/mL) and gefitinib (0.1 mol/L) significantly augmented the apoptosis in NCI-H1975 lung cancer cells by blocking EGFR signalling pathways, notably Akt and ERK1/2 phosphorylation [93]. Apart from enhancing apoptosis, the co-treatment of curcumin and gefitinib induced autophagy-related cell death, which was associated with the suppression of histone deacetylase activities and the proteasomal degradation of EGFR proteins. Interestingly, in vivo studies confirmed a decrease in tumor weight of xenograft models as compared to control, through the reduced expression of oncogenic proteins EGFR, Akt and cyclin D1, and enhanced caspase 3/8 activities, while being harmless to other tissues in xenograft models [94,95]. Similar to gefitinib, erlotinib (1 μM) elevated its apoptotic effects to PC-9 lung cancer cells when co-administered with curcumin (25 μM) through the elevation of caspase 3 activities and the downregulation of EGFR proteins. In vivo studies further confirmed that the reduction of tumor growth was associated with the downregulation of NF-κB [96,97].

Platinum-based chemotherapy, such as cisplatin and carboplatin, forms the standard chemotherapy regimen in the advanced stage of lung cancer. Despite yielding better overall survival, these treatment regimens are often associated with undesirable toxicity and the relapse of lung cancer [158]. Studies on lung cancer cells showed that curcumin treatment could lower the concentration of cisplatin needed to achieve the same cytotoxicity effect when compared to cisplatin monotherapy [98,99]. This was supported by the reduction in cisplatin-induced thymidine phosphorylase (TP) and excision repair 1, endonuclease non-catalytic subunit (ERCC1)-related signalling such as PI3K/Akt/Snail signalling [100]. Additionally, the co-treatment of curcumin and cisplatin synergistically elevated apoptotic activities in A549 lung cancer cells, mainly via the upregulation of tumor suppressor proteins p53, p21 and downregulation of oncogenic proteins EGFR, HIF-1α, NF-κB, Akt, mTOR [99,100,101]. A transwell invasion study revealed that the co-treatment of curcumin and cisplatin diminished the invasiveness of lung CSCs that are the main drivers of tumor invasion and chemoresistance [102,103]. Likewise, the decrease in invasiveness was also observed when lung cancer cells were co-treated with curcumin (10 μM) and carboplatin (50 or 100 μM), through the repression of matrix metallopeptidase 2/9 (MMP-2/9) activities [104].

Other chemotherapeutic agents were also reported to have boosted anticancer effects when co-administered with curcumin. Cell survival assays demonstrated that curcumin (30 μM) enhanced the cytotoxicity of paclitaxel (30 μM) in paclitaxel-resistant A549 and H460 lung cancer cells through the suppression of microRNA-30c-5p-mediated metastasis-associated protein 1 (MTA1), which further limited the metastasis behaviour in lung cancer cells [105]. Besides, the co-delivery of curcumin and paclitaxel encapsulated in poly (B-cyclodextrintriazine) (PCDT) exerted the synergistic inhibition of clonogenic formation and increased apoptotic events in H1299 lung cancer cells, with better solubility, bioavailability and stability provided by the PCDT delivery system [106]. Doxorubicin, which is notorious for its cardiotoxicity, is also challenged by multidrug resistance development [107]. These problems were improved with a nanomicelle delivery system encapsulating both doxorubicin and curcumin, which was responsible for promoting endocytosis and increasing drug-release capacity, while being harmless to the normal cells in vitro and in vivo [107,108,109].

### 4.4. Curcumin Combination Therapy in Pancreatic Cancer

In pancreatic cancer, the co-treatment of curcumin and gemcitabine synergistically promoted apoptosis via the downregulation of NF-κB. Furthermore, the invasiveness of pancreatic cancer cells was diminished through the downregulation of N-cadherin, vimentin and the upregulation of E-cadherin [110]. Immunohistochemistry analysis indicated the decreased expression of EMT markers MMP-9, ICAM-1 and COX-2, suggesting a reduced metastasis behaviour in xenograft models co-treated with curcumin and gemcitabine. Furthermore, the inhibition of angiogenesis was observed in xenograft models via the suppressed expression of CD31 microvessel density marker [111]. Another study demonstrated that co-treatment of curcumin (20 μM) and gemcitabine (50 nM) remarkedly impeded the formation of spheroid-derived CSCs by inhibiting the polycomb repressive complex 2 (PRC2)/plasmacytoma variant translocation 1 (PVT1)/cellular-myelocytomatosis (c-Myc) axis [112]. The high level of multidrug resistance-associated protein 5 (MRP5) in pancreatic cancer cells was profoundly repressed by the co-treatment of curcumin and 5-FU, implying that curcumin potentiates the sensitivity of 5-FU to pancreatic cancer cells [114].

Lev-Ari et al. (2005) showed that the combined treatment of curcumin (15 μM) and celecoxib (25 μM) significantly reduced the cell proliferation and enhanced apoptotic effects in P-34 pancreatic cancer cells by suppressing COX-2 expression, as compared to celecoxib monotherapy [113]. The chemoprevention of curcumin (7.5 μM) and tolfenamic acid (50 μM) synergistically stimulated the apoptotic effects in pancreatic cancer cells via the downregulation of survivin and suppression of specificity protein 1 (Sp1). Additionally, this treatment regimen induced G1 and G2 cell cycle arrest with lesser translocation of NF-κB into the nucleus, implying a diminished cell proliferation [115].

In another study, cell death analysis illustrated that the combined treatment of curcumin (10 μM) and sulforaphane (5 μM) significantly enhanced the apoptotic effect of pancreatic cancer cells treated with aspirin (1 mM). With the decrease in cell survival, this chemoprevention regimen successfully impeded Akt phosphorylation and NF-κB activity [116]. Further maximizing the therapeutic efficacy, this co-treatment regimen was encapsulated in chitosan-coated solid lipid nanoparticles for better drug delivery in xenograft models. Apart from exhibiting no toxicity, this delivery system also demonstrated slow and sustained drug release profile, and thereby significantly lessened the tumor progression in in vivo studies [117,118]. On the other hand, apoptotic activity was synergistically augmented when BxPC-3 and Panc-1 pancreatic cancer cells co-treated with garcinol and curcumin showed elevated caspase 3/9 activities [119]. In a study investigating curcuminoids emulsified in omega-3 fatty acids combined with anti-oxidant Resolvin D1, it was revealed that the combination significantly induced apoptosis in pancreatic cancer cells via the activation of caspase 3 activity. Furthermore, this combined treatment potentiated the cytotoxicity effect and inhibited interferon γ (IFNγ) production in NK cells when co-cultured with pancreatic cancer cells [120].

### 4.5. Curcumin Combination Therapy in Prostate Cancer

Although the early stage of prostate cancer can be managed well with radiation and surgery, many patients eventually progress into metastatic prostate cancer due to irresponsiveness to androgen deprivation therapy (ADT) and chemotherapy resistance. This translates to the poor prognosis of prostate cancer patients [159,160]. Despite being approved by the FDA, the combination of docetaxel with prednisone or estramustine only demonstrated modest clinical benefits to prostate cancer patients [161]. The combined treatment of curcumin (20 μM) and docetaxel (10 nM) potentiated apoptotic effects in PC3 prostate cancer cells through the downregulation of Bcl-2, Bcl-xL, and Mcl-1 and upregulation of Bak and Bid. Furthermore, the decrease in cell proliferation was correlated with the reduced CDK1, Akt, EGFR, and HER2 expression [121]. An EGFR-targeted nanoparticle delivery system containing curcumin (0.58 μM) and docetaxel (0.058 μM) was developed to induce EGFR-mediated endocytosis in prostate cancer cells [162]. Besides being stable, this delivery system also successfully reduced the tumor burden of xenograft models without causing any systemic toxicity [122].

In recent years, tumor necrosis factor-related apoptosis-inducing ligand (TRAIL) has become an attractive therapeutic agent in combating cancer through apoptosis. Current evidence showed limited positive outcomes in several clinical studies, mainly due to its poor agonist activity [163]. Despite being proven to be safe and cause no toxicity to normal tissues, TRAIL was profoundly correlated with its therapeutic resistance in cancer cells [22,164]. Therefore, a sensitizer plays an important role in overcoming TRAIL resistance. Curcumin has been shown to be able to sensitize TRAIL-resistant prostate cancer cells to TRAIL through the upregulation of death receptors death receptor 4 (DR4), death receptor 5 (DR5) and inhibited angiogenesis [164]. Similarly, another flow cytometry cell death analysis demonstrated that the combination of curcumin (10 or 25 μmol/L) and TRAIL (20 ng/mL) remarkedly induced apoptotic activities in LNCaP prostate cancer cells by downregulating NF-κB and suppressing nuclear factor of κ light polypeptide gene enhancer in B-cells inhibitor, α (IκBα) phosphorylation [125]. Another in vivo experiment exhibited that the co-treatment of curcumin (50 mg/kg) and TRAIL (3 mg/kg) caused a significant reduction in tumor burden through downregulating Akt and NF-κB expressions [126]. Interestingly, these reports highlighted no toxicity effect in preclinical models, further proving the potential of curcumin and TRAIL combination as a safe and tolerable alternative treatment for prostate cancer in future [125,126].

The work by Eslami et al. (2020) showed that the combination treatment of curcumin (25 μM) and metformin (4 mM) synergistically elevated apoptotic activities with the downregulation of mTOR activities in LNCaP prostate cancer cells [123]. In terms of combination with other natural compounds, curcumin (50 μM) and epigallocatechin gallate (EGCG) (100 μM) collectively inhibited the proliferation of PC3 prostate cancer cells by causing G2/M and S phase arrest, which was linked to an elevation of p21 protein and reduced phosphorylation of retinoblastoma (Rb) [127]. Another study involving cell death analysis demonstrated that the supplementation of curcumin (5 to 10 μM) with arctigenin (1 μM) and EGCG (40 μM) synergistically induced apoptosis in LNCaP prostate cancer cells without affecting normal epithelial cells. The apoptotic effect was enhanced by an elevation of Bax/Bcl-2 ratio and a reduction in Akt and signal transducer and activator of transcription 3 (STAT3) phosphorylation [128]. Additionally, the co-administration of curcumin (60 mg/kg) and resveratrol (5.7 mg/mL) ameliorated the tumor burden of prostate cancer xenograft models via an elevated expression of tumour suppressor proteins and anti-oxidant activities [129]. Similarly, the co-treatment of curcumin with resveratrol and ursolic acid, respectively, worked in synergy to reduce the tumour volume of xenograft models, along with the decrease in mammalian target of rapamycin complex 1 (mTORC1) and signal transducer and activator of transcription (STAT) activities [130]. The combined treatment of curcumin (8.9 μM) and quercetin (8.9 μM) reversed the hypermethylation status of androgen receptor (AR) proteins that conferred ADT resistance, by restraining the activities of DNA methyltransferase. Hence, this led to enhanced AR-mediated apoptosis in prostate cancer cells [131].

### 4.6. Curcumin Combination Therapy in Other Cancers

Curcumin synergism for cancer treatment has also been studied in various other cancers. For instance, the co-treatment of curcumin (4.32 μmol/L) and 5-FU (2.16 μmol/L) exhibited a synergistic effect on the anti-proliferation of HepG2 hepatocellular carcinoma cells via the inhibition of NF-κB translocation from cytoplasm to nucleus. Concurrently, this co-treatment also suppressed the expression of COX-2 protein, thereby disrupting the uncontrolled cell survival [132]. In another study, the combined administration of curcumin (13 μM) and celecoxib (42.8 μM) synergistically induced apoptosis in liver cancer cells by elevating caspase 3 activity. Cell proliferation assay revealed that HepG2 hepatocellular carcinoma cells also exhibited a great reduction in the expression of cell survival proteins, such as Akt, NF-κB p65 and malondialdehyde (MDA), and the inhibition of VEGF expression, implying the potentiation of anti-proliferative and anti-angiogenesis effects by curcumin and celecoxib co-treatment [133]. Zhang et al. (2018) tested the combination of curcumin (10 μM) and metformin (10 mM), and found that it induced a synergistic anti-proliferative effect on HepG2 hepatocellular carcinoma cells, without harming any normal cell lines. This anti-proliferative effect was attributed to the re-expression of tumor suppressor protein phosphatase and tensin homolog (PTEN) and p53. In vivo studies further confirmed that the co-treatment enhanced apoptosis activities via the upregulation of Bax/Bcl-2 ratio and elicited anti-angiogenesis effect via the downregulation of VEGF expression [134].

Although the incidence has declined steadily in the last few decades, gastric cancer still constitutes a global health issue as concerned by a low median survival rate of less than 12 months in gastric cancer patients [165]. Numerous studies suggested the potential of combination therapy to improve the clinical outcome of gastric cancer. Doxorubicin or etoposide repeated treatments led to the aberrant amplification of NF-κB, resulting in therapeutic resistance in gastric cancer cells. This problem was resolved by the pre-treatment of curcumin (40 μmol/L) followed by doxorubicin (0.3 μmol/L) or etoposide (20 μmol/L) administration [136]. Similarly, curcumin and doxorubicin co-treatment showed significantly more induction of apoptosis and anti-mobility behaviour of AGS gastric cancer cells as compared to monotherapy and the untreated control [135]. In vivo studies also highlighted the benefits of curcumin (74 mg/kg) and 5-FU (52 mg/kg) co-administration in slowing down the tumor growth via the reduced expression of NF-κB and COX-2, without causing any toxicity effects in other body parts of gastric cancer xenograft models [137].

Being the gold standard intravesical immunotherapy for bladder cancer, Bacillus Calmette–Guerin (BCG) treatment is shown to drive therapeutic resistance in bladder cancer cells with continuous use [166]. Curcumin (10 μmol/L) has been shown to surmount this obstacle by elevating TRAIL and DR5 expressions, and downregulating NF-κB expression when being co-treated with BCG (106 colony-forming unit (CFU)), thus enhancing extrinsic apoptotic pathways and reversing BCG therapeutic resistance in bladder cancer cells. In vivo studies further elucidated the enhancement of anticancer effects by curcumin and BCG, by inhibiting cell proliferation via the downregulation of cyclin D1 and c-Myc, suppressing angiogenesis via the suppression of VEGF, and boosting apoptosis via the downregulation of Bcl-2 and survivin in xenograft models [138]. Furthermore, the co-treatment of curcumin (10 μM) and cisplatin (10 μM) induced ROS-mediated apoptosis, which was linked to the overactivation of MAPK/ERK kinase (MEK) and ERK phosphorylation. Moreover, curcumin and cisplatin co-treatment collectively elevated tumor suppressor protein PTEN as well as p53, and downregulated the phosphorylation of STAT3 in 253J-Bv and T24 bladder cancer cells [139].

In acute lymphoblastic leukemia, curcumin (15 μM) enhanced the apoptotic effects induced by imatinib (1 μM) on SUP-B15 cells through the downregulation of the Akt/mTOR pathway and the upregulation of the Bax/Bcl-2 ratio. Furthermore, the combination treatment also inhibited the expression of breakpoint cluster region protein–acute promyelocytic leukemia (BCR/ABL), in which imatinib monotherapy was unable to do so. In vivo studies verified that the combination of curcumin (25 mg/kg) and imatinib (5 mg/kg) reduced the leukemia burden in mice with a decreased expression of BCR/ABL [140]. Further investigations illustrated that curcumin (10 μM) with other chemotherapeutic agents, notably imatinib and vincristine, synergistically induced apoptosis via the downregulation of Bcl-2 and anti-angiogenesis effect via the downregulation of VEGF in ALL cells. Besides, combination treatment reversed the NF-κB activity induced by imatinib and vincristine [141]. Similarly, curcumin (40 μM) potentiated the inhibitory effect of thalidomide (80 μM) in acute myeloid leukemia cells KG-1 and U937 by downregulating the Bcl-xL expression in apoptosis and repressing STAT3 expression [142]. Apart from that, the combination of natural products, curcumin (13.47 μM) and quercetin (53.89 μM) synergistically induced apoptosis in chronic myeloid leukemia cells K562 via the downregulation of Bcl-2 and elevated cytochrome c release to the cytosol. Furthermore, the enhancement of apoptotic effects was evident through the elevation of ROS production and loss of mitochondrial membrane potential [143].

## 5. Curcumin Combination Therapy from Bench to Bedside

Successful preclinical results may not always translate to positive clinical outcomes; hence, clinical investigations in humans are crucial. To date, numerous clinical trials on curcumin combination therapy have been carried out (Table 2), evaluating the safety and tolerability of combined treatments, toxicity profiles, and therapeutic response of patients. These studies provide imperative information for clinicians in designing newly improved robust therapeutic interventions.

Curcumin combination therapy was proven to be safe and tolerable in the clinical trials of breast cancers [167], chronic myeloid leukemia [169], colorectal cancer [88,170], pancreatic cancer [172,174] and prostate cancers [176,177]. Furthermore, patients experienced lesser toxicity effects under the curcumin combination therapy with an improved quality of life. In a phase II placebo-controlled clinical trial, metastatic breast cancer patients encountered lesser treatment-emergent adverse events (TEAEs) under the treatment of paclitaxel (80 mg/m^2^) and curcumin (300 mg solution) as compared to the patients receiving the placebo [167]. Moreover, the safety and tolerability of curcumin (up to 2 g) was well documented when co-administered with folinic acid, 5-FU and oxaliplatin (FOLFOX) chemotherapy to metastatic colorectal cancer patients [88,170]. In another randomized, double-blinded and placebo-controlled study, prostate cancer patients who received curcumin (1440 mg/day) until the completion of ADT did not experience any serious adverse effects [176]. Similarly, the supplementation of curcumin (3 g) did not result in any adverse effects to the prostate cancer patients throughout the radiotherapy [177]. These studies present the evidence of the safety and tolerability of curcumin when co-administered with conventional therapy.

In assessing the treatment response of curcumin combination therapy, a phase I dose-escalation clinical trial showed that the maximal tolerated dose of curcumin (8000 mg/day) co-treated with docetaxel (100 mg/m^2^) recorded 5/8 patients had partial response (PR) and 3/8 patients had stable disease (SD), with a significant decrease in the tumor marker carcinoembryonic antigen (CEA) and VEGF biomarkers of metastatic breast cancer [168]. Imatinib (400 mg twice per day) supplemented with turmeric powder (5 g three times/day dissolved in 150 mL of milk) achieved a higher complete remission in chronic myeloid leukemia patients. Additionally, curcumin combination treatment caused a better reduction in the nitric oxide level in patients, when compared with imatinib monotherapy [169].

In terms of the overall response rate (ORR), advanced breast cancer patients receiving curcumin (300 mg solution) and paclitaxel (80 mg/m^2^) experienced significantly higher ORR than patients receiving the placebo [167]. The co-administration of curcumin (2 g) into FOLFOX-based chemotherapy showed a higher ORR (53.3%), with a longer median progression-free survival (PFS) and overall survival (OS), as compared to the ORR of FOLFOX-based monotherapy (11.1%) in metastatic colorectal cancer patients [170]. Another clinical trial of colorectal cancer patients receiving a daily dose of curcumin (2 g) with FOLFOX chemotherapy showed 91.7% ORR, with a median PFS of 34 weeks [88]. In a phase II study on the castration-resistant prostate cancer patients treated with docetaxel (75 mg/m^2^), prednisone (5 mg) and curcumin (6000 mg/day), the ORR was 100%, with 40% having PR and 60% having SD, with a median time to progression of prostate-specific antigen (PSA) of 5.8 months [175]. Similarly, prostate cancer patients who received curcumin (1440 mg/day) in ADT had significantly lower PSA progression than patients who received ADT alone only [176]. There was an increase in plasma antioxidant capacity in prostate cancer patients who received curcumin (3 g) throughout the radiotherapy [177]. These studies demonstrated that curcumin showed treatment response when co-administrated with conventional cancer therapies.

Despite the remarkable outcomes discussed above, reports on curcumin supplementation exhibiting modest treatment response in pancreatic cancer patients have been found. In a phase II study with advanced pancreatic cancer patients treated with curcumin (8 g) and gemcitabine (1000 mg/m^2^), the ORR was reported to be 45.5%, while 54.5% of the patients had tumor progression. Besides, some patients experienced gastrointestinal toxicity, such as abdominal fullness and pain [171]. In another phase II trial, advanced pancreatic cancer patients receiving curcumin (2000 mg/die) co-treated with gemcitabine (10 mg/m^2^ or 1000 mg/mq) had about 61% of ORR; albeit, curcumin was safe and tolerable [172,174]. In another phase I/II study, gemcitabine-resistant pancreatic cancer patients who received curcumin (8 g) and gemcitabine (1000 mg/m^2^) experienced a poor 1-year survival rate [173].

Current findings confirm the effectiveness of curcumin combination treatment in breast cancer, colorectal cancer, and prostate cancer, and less significant effectiveness in pancreatic cancer. Nevertheless, a few trials on pancreatic cancers are still ongoing, with no results available to date. Hence, the conclusion can only be drawn when more data are available. At this point of time, the evidence suggests that certain cancers and/or certain cell lines are more responsive to curcumin combination anticancer treatment. This is not surprising given the different characteristics of the cancer cells, the complexities of molecular pathways involved and the variety of treatment cocktails used. Numerous clinical studies on curcumin co-administration with various conventional cancer therapy are still in progress at the time of writing (Table 3).

## 6. Research Gap and Future Directions

A large body of research demonstrated the role of curcumin in augmenting anticancer effects via combination therapy. Although the majority of studies reported positive therapeutic outcomes, some opposing results were observed in which the curcumin did not elicit a synergistic effect with combination therapy in various dosages [67,112,133]. The inconsistencies among the results warrant further investigations. To obtain robust methodology and ensure accurate results, careful considerations in the study planning is vital in both preclinical and clinical investigations.

In some studies, in vitro results were used to draw the conclusion without validation from in vivo findings. Care should be taken in interpreting these results, as in vitro findings can sometimes be a poor predictor of in vivo outcomes. Moreover, various factors, including cell-immune regulations in microenvironment and toxicity profiling, remain elusive without the integration of in vivo studies [178]. Therefore, in vivo investigations are necessary to validate the in vitro results before proceeding to human studies [41]. This means more laborious works are needed to be put in place to conduct the studies. However, it does more good than harm in the long run, with more solid preclinical evidence out there for progression into clinical trials.

Besides inducing apoptosis, curcumin is a potent regulator of autophagy in cancers. As a stress-coping mechanism, autophagy eliminates the cancer cells in the early stage of cancers. However, autophagy has also been shown to promote the survival of cancer cells by mitigating nutrient deprivation and hypoxia stress in the advanced stage of cancer [179,180]. There have been controversial opinions on whether curcumin-mediated autophagy modulates pro-survival or pro-death mechanisms in cancer cells [181]. The combination treatment of curcumin (5 μM) and berberine (25 μM) synergistically induce autophagy-related cell death in MDA-MB-231 and MCF7 breast cancer cells via the increased activation of c-Jun N-terminal kinase (JNK) signalling [76]. Similarly, the enhancement of H157 and H1299 lung cancer cell death by the combination of curcumin and gefitinib was autophagy-dependent [95]. However, Kantara et al. (2014) demonstrated that curcumin promoted autophagic cell survival of doublecortin-like kinase 1 (DCLK1) positive-CSCs in colon cancer. The ablation of DCLK1 in colon cancer CSC only restored the cell death-promoting effects mediated by curcumin, highlighting that curcumin-mediated autophagy is dependent on the expression of DCLK1 [182]. Hence, curcumin-mediated autophagy can be a double-edged sword, and this depends on cancer types and targeted signalling pathways [183,184]. This presents room for investigation to further explore the crosstalk between apoptosis and autophagy mediated by curcumin combination treatment.

Albeit curcumin is safe and tolerable [7], and the toxicity issue, especially in persistent consumption, is often overlooked due to the lack of long-term clinical studies [185]. Moreover, increasing the dosage of curcumin has been shown to negatively regulate the anti-oxidant effects by inducing DNA damage and degrading p53 proteins, leading to potential carcinogenic effects [186,187]. In vivo studies demonstrated that curcumin is an active iron chelator. This implicates that the constitutive consumption of iron will impair the iron homeostasis in patients who have a suboptimal level of iron [188]. Several in vitro studies also illustrated that curcumin and other chemotherapeutic agents exhibited antagonistic effects, reversing the anticancer effects by restricting apoptosis and cytotoxicity potential in cancer cells [189]. For instance, the curcumin dietary supplementation antagonized the cyclophosphamide-induced tumor suppression in BT474 xenograft mouse models via the downregulation of JNK activities [190]. Besides, the co-treatment of curcumin and etoposide had an antagonistic effect in MC7 breast cancer cells, HepG2 liver cancer cells, HCT116 colon cancer cells and HeLa cervical cancer cells. Cell cycle analysis revealed that this co-treatment limited the cell death of cancer cells by restricting the cells entering M phase [191]. These issues warrant the need for stringent dose–response evaluation, attentive choices of drug combination, and long-term clinical follow up to determine the optimum dose of curcumin in maximizing therapeutic response without instigating any toxicity events.

Due to the heterogeneity of cancer and disparity in the genetic makeup of cancer patients, there could be discrepancies in the therapeutic efficacy of curcumin combination therapy among cancer patients. To address this issue, precision medicine can be applied to understand the underlying cause of cancer in a patient. This can be achieved through a multitude of drug screenings using curcumin as a main therapeutic agent combined with an array of conventional therapeutic agents. In addition to that, gene sequencing technology can be a useful tool in identifying tumor-associated vulnerabilities as therapeutic targets in cancer patients. Once a specific gene mutation is confirmed, the anticancer treatment can then be designed and tailored for the patient. Undoubtly, these interventions will foster the developments of highly effective curcumin combination therapy regimens that will target the precise cause of disease in an individual patient [192,193].

It is noteworthy that in most of the clinical trials, a remarkably high dose of curcumin was used. Curcumin is notorious for its hydrophobic nature, thereby restricting its pharmacokinetic potential by having poor aqueous solubility, poor bioavailability, and rapid systemic elimination from the body [194]. These characteristics have impeded its ability to reach the target area. As a result, a high dose of curcumin has to be administered to account for these shortcomings, in order to maintain the effective plasma concentration in the blood. In reality, a high dose of curcumin might be impractical in a clinical setting, as it necessitates the administration of several large tablets/capsules for a single dose. Moreover, the gastrointestinal side effect observed in one of the clinical trials signifies the potential irritant effect of large dose of curcumin. Hence, room for improvement exists in delivering curcumin as combination therapy for cancer patients. Specifically, this issue should be addressed before this treatment option is made available to patients. In this regard, refining the formulation of curcumin appears to be an important step. The improved formulation of curcumin should address the poor bioavailability and rapid degradation issues. The nanoformulation of curcumin is one of the options to be used, as these have been shown to be able to increase drug payload in a single dose, at the same time allowing the flexibility of modifying the nanoformulation according to needs. With the proper choice of excipients, the stability of curcumin could also be improved, where the nanomaterials could protect the compound from degradation. In fact, several reports illustrated that the application of nanoformulation in curcumin combination therapy has successfully demonstrated the enhanced aqueous solubility for better delivery [195], reversing multidrug resistance [108] and ensuring the distinctive biodistribution of therapeutic agents [107,196]. On the other hand, the nanoformulation of curcumin could also be designed to target a specific site of the body, to deliver an even higher dose of the compound to the site of action, instead of distributing to other organs. One of the examples is curcumin nanoparticles with a mucoadhesive effect for colorectal cancer [196,197,198]. It is expected that the nanoparticles would have prolonged contact time with the colon due to mucoadhesion between the nanoparticles and the colonic mucin. This will allow a greater amount of curcumin to be released on-site and act on the cancer cells. Utilizing the same concept, a nanoformulation containing both curcumin and the chemotherapeutic agent can then be engineered for such delivery for better treatment outcomes. It would be interesting to observe the outcome of the ongoing clinical trials on the combination chemotherapy with curcumin nanoformulations.

## 7. Conclusions

The search for an effective cancer therapeutic strategy remains a great challenge for the scientific community due to various side effects, general cytotoxicity to cancer and normal cells, and the development of therapeutic resistance, which often lead to therapeutic failure. Combination therapy presents a good option to alleviate the tumor burdens by lowering toxicity and simultaneously targeting multiple mechanisms that modulate tumor development, without harming the healthy cells.

Curcumin holds a great promise in the development of combination therapy, which is frequently paired with conventional chemotherapeutic drugs as well as other natural compounds. An enormous body of preclinical and clinical evidence had entailed the potential of curcumin in preventing the exacerbation of cancer development by modulating multiple signalling pathways when combined with other therapeutic agents. Apart from diminishing cell survival and elevating cancer cell death, curcumin combination therapy has a robust effect in alleviating the hallmarks of cancer, such as metastasis and angiogenesis progression. The combined treatment of curcumin with other conventional therapy could overcome the pitfalls contributed by persistent conventional treatments and the genetic constitution of cancer cells, resulting in the improvement of therapeutic outcome. However, the poor pharmacokinetic issues of curcumin need to be addressed first. This could be resolved through the application of appropriate drug delivery systems, such as nanoformulation, that could effectively improve the delivery of curcumin to the target sites. The controversy surrounding the use of curcumin, such as its antagonistic effect, autophagy modulation, and potential toxicity associated with its long term use, warrants more studies and long-term clinical monitoring. With the continuous exploration of curcumin combination therapy and a deep understanding of its modulation in anticancer mechanisms, curcumin combination therapy holds great promise as a new therapeutic approach in combating cancer, especially in cancer patients irresponsive to single conventional chemotherapy. It is noteworthy that deep molecular profiling is essential for understanding the tumor-associated biomarkers that are crucially influenced by the action of curcumin, thereby shaping the future combined therapeutic strategies that eventually will translate into better oncologic outcome.

## Figures and Tables

**Table 1:** Preclinical findings of curcumin combination therapy in enhancing anticancer effects. The findings were summarized in the studies of cell line (in vitro), animal (in vivo) and tissues/organs experimentation under more controlled condition (ex vivo).

Cancer Type	Chemotherapeutic Drugs	Main Findings		References
		In Vitro	In Vivo/Ex Vivo	
Breast cancer	Tamoxifen	Inhibition of cell proliferation via the repression of Akt/mTOR signalling. Increased apoptotic activities via the activation of pro-apoptotic protein BcL-xL; repression of anti-apoptotic protein Bcl-2. Induction of G2/M arrest via the inhibition of c-Myc and cyclin D1; downregulation of p21. Reversal of tamoxifen-induced chemoresistance via the suppression of p65 phosphorylation, EZH2 expression and SLUG/HK2 pathway.		[65,66]
	Trastuzumab	Inhibition of cell proliferation via the repression of NF-κB and HER2. Induction of G2/M arrest via the repression of Akt and MAPK phosphorylation. Reduction in cancer cell migration.	Inhibition of tumor growth.	[67]
	Paclitaxel	Inhibition of cell proliferation via the suppression of COX-2 and c-Myc. Increased apoptotic activities via the activation of pro-apoptotic protein Bax; repression of anti-apoptotic protein Bcl-2; upregulation of caspase 3/8 activity. Reduction in cancer cell migration via the suppression of MMP-9 and ICAM-1. No toxicity to normal cell lines. Reversal of paclitaxel-induced chemoresistance via the upregulation of p53; downregulation of NF-κB, COX-2 and EGFR.	Inhibition of tumor growth via the suppression of NF-κB. Prevention of breast cancer metastasis to lung tissues via the downregulation of MMP-9.	[68,69,70,71]
	Doxorubicin	Inhibition of cell proliferation via the suppression of PI3K/Akt, GSK3β, β-catenin phosphorylation. Inhibition of efflux function of ABCB4 via the inhibition of ATPase activities of ABCB4. Inhibition of EMT via the upregulation of E-cadherin; downregulation of Smad2 phosphorylation.		[72]
	Doxorubicin (with curcumin loaded in solid lipid nanoparticle)	Increase intracellular retention of doxorubicin. Reversal of Pgp-induced chemoresistance via the upregulation of p50, p65 and c-Rel expression.	Inhibition of tumor growth via the suppression of p38 activation. Decreased in weight of tumor mass.	[73]
	Quercetin, Optiberry, lapatinib	Inhibition of cell proliferation via the suppression of Akt phosphorylation. Reversal of HER2-induced chemoresistance via the downregulation of HER2.		[74]
	EGCG	Increased cytotoxicity. Induction of G2/M arrest.	Reduced tumor weight and volume via the downregulation of EGFR, Akt and VEGFR-1. Co-treatment was non-toxic to mice.	[75]
	Berberine	Inhibition of cell proliferation via the activation of ERK pathway. Increased apoptotic activities via the activation of pro-apoptotic protein Bax; repression of anti-apoptotic protein Bcl-2; upregulation of caspase 3 activity and PARP cleavage. Induction of autophagic cell death via the upregulation of JNK signalling.		[76]
Colorectal cancer/colon cancer	5-FU	Inhibition of cell proliferation via the suppression of Wnt signalling. Increased apoptotic activities via the upregulation of caspase 3/8 activities and PARP cleavage; repression of Bcl-xL. Induction of G0/G1 phase and S phase arrest via the suppression of cyclin D1. Reduction in cancer cell migration and invasiveness via the suppression of MMP-9, CXCR4. Reversal of 5-FU-induced chemoresistance via the suppression of NF-κB and Pgp. Inhibition of autophagy via the downregulation of AMPK signalling.	Inhibition of tumor growth. Co-treatment was non-toxic to mice.	[77,78,79,80,81]
	Oxaliplatin	Inhibition of cell proliferation via the inhibition of TGF-β/Smad2/Smad3 signalling. Increased apoptotic activities via the upregulation of caspase 3 activity; repression of anti-apoptotic protein Bcl-2. Inhibition of EMT via the downregulation of N-cadherin; upregulation of E-cadherin.	Reduced tumor weight and volume via the suppression of Smad2 and Smad3 phosphorylation in tumor tissues.	[82]
	Irinotecan	Inhibition of cell proliferation. Increased endoplasmic reticulum stress-induced apoptotic activities via the activation of pro-apoptotic protein Bax; repression of anti-apoptotic protein Bcl-2; suppression of CHOP activity; induction of ROS production; upregulation of caspase 3/8 activity. Induction of S phase arrest. Reduced spheroid formation of cancer stem cells via the suppression of CD44, CD24 and EpCAM expression.		[83,84]
	Dasatinib	Inhibition of cell proliferation. Reduced sphere-forming potential. Reduction in cancer cell invasiveness. Decreased expression of cancer stem cell markers via the suppression of ALDH, CD44, CD133 and CD166.	Reduction in the cancer stem cells markers ALDH, CD44, CD133 and CD166 in tumor tissues.	[85]
	5-FU, oxaliplatin	Inhibition of cell proliferation via the suppression of Akt pathway. Reversal of 5-FU and oxaliplatin-induced chemoresistance via the downregulation of EGFR, HER2, HER3 and COX-2 expression.	Decreased in the number of spheroid formation via the downregulation of pluripotent stem cell markers Oct-3/4, AFP, Nanog and Otx2. Reduction in proliferation and increased apoptotic activities in ex vivo culture.	[86,87,88]
	FOLFOX	Inhibition of cell proliferation via the suppression of stem cell markers CD166, CD44 and EGFR expression in FOLFOX-surviving cancer cells. Increased the methylation status of EGFR. Suppression of EGFR expression via the upregulation of DNMT1 and methylation status of EGFR. Inhibition of colony formation.		[89]
	FOLFOX, bevacizumab		Inhibition of tumor growth via the increase in apoptosis in tumor area. Co-treatment was non-toxic to mice. Reduction in the angiogenesis effect by reduced expression of CD31 and Factor VIII in stained tissues.	[90]
	Silymarin	Inhibition of cell proliferation. Increased apoptotic activities via the upregulation of caspase 3/7 activities.		[91]
	Resveratrol	Inhibition of cell proliferation via the suppression of NF-κB, EGFR, HER2, HER3 and IGF-1R. Increased apoptotic activities. Induction of S phase arrest.	Inhibition of tumor growth via the inhibition of mitotic index and increased apoptosis in tumor area. Co-treatment was non-toxic to mice.	[92]
Lung cancer	Gefitinib	Inhibition of cell proliferation via the suppression of Akt and ERK1/2 phosphorylation; induction of proteasomal-induced degradation of EGFR protein; inhibition of EGFR pathway. Increased apoptotic activities via the upregulation of caspase 3 activity and PARP cleavage; repression of anti-apoptotic protein survivin. Induction of autophagy-related cell death via the increase in LC3-II. Harmless to intestinal epithelial cell line.	Inhibition of tumor growth. Reduction in tumor volume via the downregulation of Sp1/EGFR signalling. Attenuation of adverse gastrointestinal effects induced by gefitinib.	[93,94,95]
	Erlotinib	Inhibition of cell proliferation via the downregulation of NF-κB and EGFR; upregulation of IκB expression. Increased apoptotic activities via the upregulation of caspase 3/9 cleavage; repression of anti-apoptotic protein survivin.	Inhibition of tumor growth via the upregulation of IκB; downregulation of NF-κB. Reduction in tumor weight. No metastases were observed in tissues.	[96,97]
	Cisplatin	Inhibition of cell proliferation via the downregulation of cyclin D1, Akt/mTOR pathway; upregulation of p21. Increased apoptotic activities via the activation of pro-apoptotic protein Apaf-1; repression of anti-apoptotic protein Bcl-2; upregulation of caspase 3/9 activity. Inhibition of cancer cell migration. Reversal of cisplatin-induced chemoresistance via the downregulation of FANCD2 monoubiquitination, ERCC1-related signalling pathways, CA916798 expression and HIF-1α expression through ubiquitin–proteasome degradation.	Inhibition of tumor growth. Reduction in tumor volume.	[98,99,100,101,102]
	Cisplatin, radiotherapy	Inhibition of cell proliferation via the suppression of EGFR expression. Inhibition of cancer cell migration and invasiveness. Sensitization to radiotherapy treatment via the suppression of EGFR expression.		[103]
	Carboplatin	Inhibition of cell proliferation via the upregulation of p21 and ERK1/2 phosphorylation; downregulation of NF-κB expression and Akt phosphorylation. Increased apoptotic activities via the activation of pro-apoptotic proteins Bax and p53; suppression of anti-apoptotic protein Bcl-2. Inhibition of cancer cell migration and invasiveness via the downregulation of MMP-2/9 activity.		[104]
	Paclitaxel	Inhibition of cell proliferation. Reversal of paclitaxel-induced chemoresistance via the downregulation of miR-30c-5p-directed MTA1 mRNA.		[105]
	Paclitaxel (with curcumin encapsulated in PCDT)	Inhibition of cell proliferation. Inhibition of clonogenic formation. Increased apoptotic activities.		[106]
	Doxorubicin (with curcumin loaded with micelles)	Maintained distinctive biodistribution profile of doxorubicin. Micelle system remained stable for 24 h. Harmless to normal cell line.	Inhibition of tumor growth. Reduction in cardiotoxicity effect induced by doxorubicin via the downregulation of SOD and GSH-Px. Body tissues were protected from oxidative stress.	[107]
	Doxorubicin (with curcumin loaded in nanomicelles)	Inhibition of cell proliferation via the suppression of Pgp function. Increased cellular uptake of doxorubicin and curcumin via endocytosis. Harmless to normal cell line.	Inhibition of tumor growth.	[108]
	Doxorubicin (with curcumin encapsulated in U-11 targeting nanoparticle)	Inhibition of cell proliferation. Increased cellular uptake of doxorubicin and curcumin.	Inhibition of tumor growth.	[109]
Pancreatic cancer	Gemcitabine	Inhibition of cell proliferation. Increased apoptotic activities via the upregulation of caspase 3 activity and PARP cleavage; repression of anti-apoptotic protein Bcl-2, Bcl-xL and IAP-1. Inhibition of cancer cell migration and invasiveness via the upregulation of E-cadherin; downregulation of N-cadherin and vimentin; downregulation of MMP-2/9 activities. Induction of G2/M arrest via the upregulation of p21; suppression of PCNA. Inhibited the spheroid-derived cancer stem cell formation via the suppression of PRC2/PVT1/c-Myc pathways.	Inhibition of tumor growth via the downregulation of Ki67, NF-κB, COX-2, cyclin D1. Reduction in tumor volume and weight. Inhibition of angiogenesis via the downregulation of microvessel density marker CD31 and VEGF.	[110,111,112]
	Celecoxib	Inhibition of cell proliferation via the downregulation of COX-2. Increased apoptotic activities.		[113]
	5-FU	Inhibition of cell proliferation. Reversal of 5-FU-induced chemoresistance via the inhibition of drug efflux function mediated by MRP5.		[114]
	Aspirin, sulforaphane	Inhibition of cell proliferation via the upregulation of ERK1/2 phosphorylation, p53 and p38 MAPK proteins; suppression of Akt phosphorylation; downregulation of NF-κB activity. Increased apoptotic activities via the upregulation of caspase 3 and PARP cleavage.		[115]
	Aspirin, sulforaphane (with curcumin encapsulated in chitosan coated solid lipid nanoparticle)		Encapsulation system induced slow and sustained drug release. Increased cellular uptake of curcumin, aspirin and sulforaphane into lysosome. Reduction in tumor growth. Co-treatment was non-toxic to mice, especially to kidney, heart, liver and brain.	[116,117]
	Tolfenamic acid	Inhibition of cell proliferation. Increased apoptotic activities via the upregulation of caspase 3 activity, ROS production and PARP cleavage; repression of anti-apoptotic protein survivin. Induction of G0/G1 and G2/M arrest via the downregulation of NF-κB translocation to nucleus.		[118]
	Garcinol	Inhibition of cell proliferation. Increased apoptotic activities via the upregulation of caspase 3/9 activity.		[119]
	Omega-3 fatty acid, anti-oxidant Resolvin D1	Increased apoptotic activities via the upregulation of caspase 3 activity. Increased cytocidal effect of pancreatic cancer cells by NK cells via the production of IFN-γ.		[120]
Prostate cancer	Docetaxel	Inhibition of cell proliferation via the downregulation of NF-κB, COX-2, Akt, EGFR, HER2 and CDK-1. Increased apoptotic activities via the activation of pro-apoptotic protein Bak and Bid; repression of anti-apoptotic protein Bcl-2, Bcl-xL and Mcl-1.		[121]
	Docetaxel (with curcumin encapsulated in EGFR peptide targeted, pH sensitive nanoparticle)	Nanoparticles remained stable and had better cumulative drug release in pH 5.0 as compared to pH 7.4. Increased cellular uptake of curcumin and docetaxel via EGFR-mediated endocytosis. Inhibition of cell proliferation.	Inhibition of tumor growth. Co-treatment was non-toxic to mice.	[122]
	Metformin	Inhibition of cell proliferation via the downregulation of mTOR and hTERT. Increased apoptotic activities via the activation of pro-apoptotic protein Bcl-xL; repression of anti-apoptotic protein Bcl-2.		[123]
	TRAIL	Inhibition of cell proliferation via the downregulation of Akt, NF-κB and IκBα phosphorylation. Increased apoptotic activities via the upregulation of caspase 3/8 activities; upregulation of DR4 and DR5. Inhibition of angiogenesis via the downregulation of ERK signalling.	Inhibition of tumor growth via the downregulation of NF-κB and Akt phosphorylation. Increased apoptotic activities in tumor tissues via the detection of high number of TUNEL positive cells.	[124,125,126]
	EGCG	Inhibition of cell proliferation. Induction of S and G2/M arrest via the upregulation of p21; downregulation of Rb phosphorylation.		[127]
	EGCG, arctigenin	Inhibition of cell proliferation via the suppression of STAT3, NF-κB and Akt phosphorylation. Increased apoptotic activities via the activation of pro-apoptotic protein Bax; repression of anti-apoptotic protein Bcl-2. Induction of G0/G1 arrest. Inhibition of cancer cell migration. Harmless to normal epithelial cells.		[128]
	Resveratrol		Inhibition of tumor growth. Increased apoptotic activities. Increase in anti-oxidant activities via the upregulation of GSH, SOD, GST and GR activities.	[129]
	Resveratrol, ursolic acid	Inhibition of cell proliferation via the suppression of ATP activities; the release of ROS.	Inhibition of tumor growth via the downregulation of Src, mTORC1 and STAT3 phosphorylation; suppression of AMPK activation. Decrease in tumor volume and weight of mice.	[130]
	Quercetin	Inhibition of cell proliferation via the upregulation of p21 and p27. Increased apoptotic activities via the activation of pro-apoptotic protein Bax; upregulation of caspase 3 activity. Reversal of androgen deprivation therapy-induced chemoresistance via the upregulation of AR protein; downregulation of DNMT activity.		[131]
Liver cancer	5-FU	Inhibition of cell proliferation via the suppression of NF-κB translocation from cytoplasm to nucleus; downregulation of COX-2 expression.	Inhibition of tumor growth. Co-treatment was non-toxic to mice.	[132]
	Celecoxib	Inhibition of cell proliferation via the downregulation of NF-κβ, PGE2, MDA and Akt phosphorylation; suppression of cyclin D1 and VEGF expression. Increased apoptotic activities via the upregulation of caspase 3 activity.		[133]
	Metformin	Inhibition of cell proliferation via the upregulation of PTEN and p53; downregulation of NF-κB, Akt and mTOR phosphorylation. Harmless to normal cell lines. Increased apoptotic activities via the repression of anti-apoptotic protein Bcl-2; activation of pro-apoptotic protein Bax; upregulation of PARP cleavage. Inhibition of cancer cell migration and invasiveness via the downregulation of VEGF and EGFR proteins. Inhibition of angiogenesis via the reduction in the number of HUVEC.	Inhibition of tumor growth. Increased apoptotic activities via the elevation of Bax/Bcl-2 ratio. Inhibition of angiogenesis via the reduction in VEGF expression.	[134]
Gastric cancer	Doxorubicin	Inhibition of cell proliferation. Increased apoptotic activities via the activation of pro-apoptotic protein Bax; repression of anti-apoptotic protein Bcl-2; upregulation of caspase 9 activity. Inhibition of cancer cell migration and invasiveness.		[135]
	Doxorubicin, etoposide	Inhibition of cell proliferation via the repression of anti-apoptotic protein Bcl-2. Reversal of doxorubicin and etoposide-induced chemoresistance via the suppression of NF-κB and IκBα degradation.		[136]
	5-FU	Inhibition of cell proliferation via the suppression of COX-2 and NF-κB.	Inhibition of tumor growth. Co-treatment was non-toxic to mice.	[137]
Bladder cancer	BCG	Inhibition of cell proliferation. Increased apoptotic activities via the upregulation of DR5 and TRAIL/Apo2L. Reversal of BCG-induced chemoresistance via the suppression of NF-κβ.	Inhibition of tumor growth via the suppression of NF-κB-related gene products such as cyclin D1 and c-Myc. Inhibition of tumor volume via the suppression of proliferation marker Ki67 and microvessel density marker CD31 and VEGF. Increased apoptotic activities via the suppression of anti-apoptotic protein Bcl-2 and survivin; upregulation of pro-apoptotic protein Bcl-xL.	[138]
	Cisplatin	Inhibition of cell proliferation via the upregulation of ERK and MEK phosphorylation; upregulation of p53 and p21; elevation of STAT3 phosphorylation. Increased apoptosis via the production of ROS; repression of anti-apoptotic protein Bcl-2 and XIAP. Inhibition of cancer cell migration.	Inhibition of tumor growth. Co-treatment was non-toxic to mice.	[139]
Acute lymphoblastic leukemia	Imatinib	Inhibition of cell proliferation via the downregulation of Akt/mTOR pathway and suppression of BCR/ABL. Increased apoptosis via the repression of anti-apoptotic protein Bcl-2; activation of pro-apoptotic protein Bax. Harmless to normal cell lines.	Inhibition of tumor growth via the suppression of BCR/ABL. Reduction in leukemia burden via the reduction in leukemic infiltration into the spleen.	[140]
	Imatinib, vincristine	Inhibition of cell proliferation via the suppression of VEGF and CCND1. Reversal of imatinib and vincristine-induced chemoresistance via the downregulation of NF-κB. Increase apoptosis via the repression of anti-apoptotic protein Bcl-2.		[141]
Acute myeloid leukemia	Thalidomide	Inhibition of cell proliferation via the downregulation of STAT3. Increased apoptosis via the repression of anti-apoptotic protein Bcl-xL.		[142]
Chronic myeloid leukemia	Quercetin	Inhibition of cell proliferation. Increased apoptosis via the repression of anti-apoptotic protein Bcl-xL; upregulation of PARP cleavage; upregulation of caspase 9 activity; elevation in cytochrome *c* release; enhanced ROS production; elevated loss of mitochondrial membrane potential; decrease in intracellular GSH.		[143]

Note: 5-FU, 5-fluorouracil; ABCB4, ATP binding cassette subfamily B member 4; AFP, alpha-fetoprotein; Akt, protein kinase B; ALDH, aldehyde dehydrogenase; AMPK, AMP-activated protein kinase; Apaf-1, apoptotic protease activating factor-1; Apo2L, Apo2 ligand; AR, androgen receptor; ATP, adenosine triphosphate; Bak, Bcl-2 homologous antagonist killer; Bax, Bcl-2-associated X protein; BCG, Bacillus Calmette–Guerin; Bcl-2, B-cell lymphoma 2; Bcl-xL, B-cell lymphoma-extra-large; BCR/APL, breakpoint cluster region protein-acute promyelocytic leukemia; Bid, BH3-interacting domain death agonist; CCND1, cyclin D1; CDK-1, cyclin-dependent kinase 1; CHOP, C/EBP homologous protein; c-Myc, cellular-master regulator of cell cycle entry and proliferative metabolism; COX-2, cyclooxygenase-2 CXCR4, C-X-C chemokine receptor type 4; DNMT, DNA methyltransferase; DNMT1, DNA methyltransferase 1; DR4, death receptor 4; DR5, death receptor 5; EGCG, epigallocatechin gallate; EGFR, epidermal growth factor receptor; EMT, epithelial-to-mesenchymal transition; EpCAM, epithelial cell adhesion molecule; ERCC1, DNA excision repair protein; ERK, extracellular-signal-regulated kinase; ERK1, extracellular-signal-regulated kinase 1; ERK2, extracellular-signal-regulated kinase 2; FANCD2, Fanconi anemia group D2; FOLFOX, folinic acid, 5-fluorouracil and oxaliplatin; GR, glutathione reductase; GSH, glutathione; GSH-Px, glutathione peroxidase; GSK3β, glycogen synthase kinase 3 β; GST, glutathione S-transferase; HER2, human epidermal growth factor receptor 2; HER3, human epidermal growth factor receptor 3; HIF-1α, hypoxia-inducible factor 1-α; HK2, hexokinase 2; hTERT, human telomerase reverse transcriptase; HUVEC, human umbilical vein endothelial cells; IAP-1, inhibitor of apoptosis-1; ICAM-1, intercellular adhesion molecule 1; IFN-γ, interferon-γ; IGF-1R, insulin-like growth factor 1 receptor; IκB, nuclear factor of κ light polypeptide gene enhancer in B-cells inhibitor; IκBα, nuclear factor of κ light polypeptide gene enhancer in B-cells inhibitor, α; JNK, c-Jun N-terminal kinase; Ki67, marker of proliferation; LC3-II, Microtubule-associated protein 1A/1B-light chain 3- phosphatidylethanolamine conjugate; MAPK, mitogen-activated protein kinase; Mcl-1, myeloid cell leukemia-1; MDA, malondialdehyde; MEK, mitogen-activated protein kinase/ERK kinase; MMP-2, matrix metallopeptidase 2; MMP-9, matrix metallopeptidase 9; MRP5, multidrug resistance-associated protein 5; MTA1, metastasis associated 1; mTOR, mammalian target of rapamycin; mTORC1, mammalian target of rapamycin complex 1; Nanog, homeobox protein; NF-κB, nuclear factor κ-light-chain-enhancer of activated B cells; NK, natural killer cells; Oct-3, octamer-binding transcription factor 3; Oct-4, octamer-binding transcription factor 4; Otx2, orthodenticle homeobox 2; PARP, poly (ADP-ribose) polymerase; PCDT, poly (β-cyclodextrin triazine); PGE2, prostaglandin E_2_; Pgp, P-glycoprotein 1; PI3K, phosphoinositide 3-kinase; PRC2, polycomb repressive complex 2; PTEN, phosphatase and tensin homolog; PVT1, plasmacytoma variant translocation 1; Rb, retinoblastoma; ROS, reactive oxygen species; SLUG, Snail-related zinc-finger transcription factor; SOD, superoxide dismutase; Sp1, specificity protein 1; Src, proto-oncogene tyrosine-protein kinase; STAT3, signal transducer and activator of transcription 3; TGF-β, transforming growth factor-β; TRAIL, tumor necrosis factor-related apoptosis inducing ligand; TUNEL, terminal deoxynucleotidyl transferase dUTP nick end labelling; VEGF, vascular endothelial growth factor; VEGFR-1, vascular endothelial growth factor receptor-1; Wnt, wingless-related integration site.

**Table 2:** Completed clinical trials in curcumin combination therapy.

Clinical Trials Identifier	Clinical Trials	Phase	Cancer Type	Treatment Regimens	Objective and Findings	Reference
NCT03072992	Efficacy and safety of curcumin in combination with paclitaxel in patients with advanced, metastatic breast cancer: A comparative, randomized, double-blind, placebo-controlled clinical trial	2	Breast cancer	Curcumin group: 8 mg dexamethasone, curcumin (CUC-01, 300 mg solution) and paclitaxel (80 mg/m^2^) injected intravenously Placebo group: 8 mg dexamethasone, placebo and paclitaxel (80 mg/m^2^) injected intravenously.	Curcumin group (51%) had significantly higher ORR than placebo group (31%). The efficacy was maintained for more than 3 months in curcumin group, with higher RECIST score than baseline. Median PFS in curcumin group (27.0 weeks) was 2.4 weeks longer than placebo group (24.6 weeks). TEAEs were lessreported in curcumin group as compared to placebo group.	[167]
NA	Phase I dose escalation trial of docetaxel plus curcumin in patients with advanced and metastatic breast cancer	1	Breast cancer	Docetaxel (100 mg/m^2^) as intravenous infusion on day 1 of each 3 week cycle for 6 cycles. Premedicated with 50 mg BID of oral methylprednisolone given two days before and after chemotherapy. Six dose levels of curcumin (500 mg/day) for consecutive 7 days at each cycle.	Maximal tolerated dose of curcumin was at 8000 mg/day. Out of 8 patients, 5 patients had PR and 3 patients had SD. Tumor marker CEA decreased significantly in patients with PR and SD from the 3rd cycle of treatment. VEGF significantly decreased by 30% between baseline and cycle no. 3, and by 21% between baseline and cycle no. 6.	[168]
NA	Effect of imatinib therapy with and without turmeric powder on nitric oxide levels in chronic myeloid leukemia	NA	Chronic myeloid leukemia	Curcumin group: imatinib (400 mg twice a day) along with turmeric powder (5 g three times/day dissolved in 150 mL of milk to improve its platability and absorption) for 6 weeks. Imatinib group: imatinib (400 mg twice a day for 6 weeks).	Curcumin group achieved larger percentage of complete remission, with no significant difference with imatinib group. Curcumin group (4.06 ± 1.79 μmol/L) has better reduced nitric oxide level than imatinib group (14.26 ± 276 μmol/L), but both nitric oxide levels were significantly reduced as compared to inital nitric oxide level, 42.43 ± 5.79 μmol/L). Limited common side effects were observed.	[169]
NCT01490996	Curcumin Combined with FOLFOX Chemotherapy Is Safe and Tolerable in Patients with Metastatic Colorectal Cancer in a Randomized Phase IIa Trial	2a	Colorectal cancer	FOLFOX: FOLFOX ± bevacizumab. CUFOX: FOLFOX ± bevacizumab plus 2 g oral Curcumin C3 complex per day. Chemotherapy was given once every 2 weeks for ≤12 cycles or until patient progression, unacceptable toxicity, death or withdrawal.	CUFOX was safe and well tolerated. At 12 cycle of treatment, ORR reached 11.1% and 53.3% for FOLFOX and CUFOX. Median PFS of CUFOX (320 days) was higher than FULFOX (171 days). Median OS of CUFOX (596 days) was higher than FULFOX (200 days). CXCL1 in explant culture treated with CUFOX (180 pg/mL) was lower than treated with FOLFOX (370 pg/mL). No significant difference between arms for QoL or neurotoxicity.	[170]
NCT01490996	Curcumin inhibits cancer stem cell phenotypes in ex vivo models of colorectal liver metastases, and is clinically safe and tolerable in combination with FOLFOX chemotherapy	1	Colorectal cancer	Daily curcumin dose (0.5, 1 and 2 g) 7 days prior the chemotherapy. FOLFOX-based chemotherapy was 2-weekly cycles given to a maximum of 12 cycles or until withdrawal from the trial.	Cur is safe and tolerable up to 2 g daily. 91.7% patients showed SD or PR to treatment. Median PFS was 34 weeks.	[88]
NCT01859858	Effect of Curcumin on Dose Limiting Toxicity and Pharmacokinetics of Irinotecan in Patients With Solid Tumors	1	Colorectal cancer	Experimental group 1: oral curcumin (1 to 4 g/day) for 4 days prior to irinotecan + 200 mg/m^2^ irinotecan intravenous injection, days 1 and 15 Experimental group 2: maximal tolerated dose of oral curcumin as determined from Experimental group 1 + 200 mg/m^2^ irinotecan intravenous injection, days 1 and 15	To determine the safety, pharmacokinetics and effectiveness of irinotecan when given in combination with curcumin in patients with metastatic colorectal cancer. To better understand the interaction between curcumin and irinotecan by measuring levels of irinotecan in blood when a patient also takes curcumin. Information will result in improved dosing guidelines and lead to more effective treatment with lesser toxicity.	NA
NCT00192842	Curcumin and Gemcitabine in Patients With Advanced Pancreatic Cancer	2	Pancreatic Cancer	A total of 8 g of curcumin (Sabinsa Corporation) by mouth daily concurrently with gemcitabine (1000 mg/m^2^) intravenously weekly for 3 of 4 weeks.	Some gastrointestinal toxicity such as abdominal fullness and pain were observed appeared in 7 patients. Local control rate of 45.5% was recorded, with 9.1% of patients having partial response for 7 months; 36.4% of patients had stable disease lasting for 2, 3, 6, and 12 months; 54.5% of patients had tumor progression. The median TTP was 2.5 months. The median OS was 5 months.	[171]
NA	Phytosome complex of curcumin as complementary therapy of advanced pancreatic cancer improves safety and efficacy of gemcitabine: Results of a prospective phase II trial	2	Pancreatic Cancer	Meriva (curcumin) 2000 mg/die continuously (4 capsules, each 500 mg, every day) and gemcitabine (10 mg/m^2^/min infused over 100 min and diluted in 500 mL of normal saline on days 1, 8, 15 in the dose-intense schedule. Each cycle was given every 28 days.	A percentage of 27.3% of RR and 34.1% of SD, totalizing a disease control rate of 61.4%. Median PFS and OS were 8.4 and 10.2 months, respectively. Curcumin is safe and efficiently translates in a good RR in first line therapy of advanced pancreatic cancer.	[172]
UMIN-ID 000001386	A phase I/II study of gemcitabine-based chemotherapy plus curcumin for patients with gemcitabine-resistant pancreatic cancer	1 and 2	Pancreatic Cancer	Curcumin group: oral curcumin (8 g) + intravenous administration of gemcitabine at a dose of 1000 mg/m^2^ on days 1 and 8 and 60 mg/m^2^ of S-1 orally for 14 consecutive days every 3 weeks. Experimental group: gemcitabine monotherapy	Advanced pancreatic cancer patients completed first cycle treatment for phase 1 study without any dose limited toxicity at 8 g/day. Median OS time was 161 days and 1 year survival rate was 19%. Oral curcumin with 8 g was safe and feasible in patients with advanced pancreatic cancer.	[173]
NA	Phase II study of gemcitabine and curcumin as first line treatment for locally advanced or metastatic pancreatic cancer: preliminary data	2	Pancreatic Cancer	Gemcitabine (1000 mg/mq in 100 min on day 1, 8, 15 every 28 days) and curcumin (2000 mg/day, continuously) until progression or unacceptable toxicities or patients refusal.	Overall RR was 28.2% and SD was 33.3% of cases, totalizing a disease control rate of 61.5%. Supplementation of curcumin to gemcitabine was safe and well tolerated.	[174]
NCT01012141	The New Combination Docetaxel, Prednisone and Curcumin in Patients with Castration-Resistant Prostate Cancer: A Pilot Phase II Study	2	Prostate Cancer	Curcumin 6000 mg per day (12 capsules with 500 mg in each capsule) for 7 consecutive days in each cycle + prednisone 5 mg or prednisoline orally twice daily on day 1 + docetaxel, 75 mg/m^2^ delivered as a 1 h intravenous infusion on day 1 every 21 days for 6 cycles, with pre-medication with dexamethasone, 8 mg given 12, 3 and 1 h before docetaxel infusion.	A total of 34% of patients had stable PSA levels and 7% presented with PSA progression. A total of 40% had partial response, 60% had stable disease; all patients benefit from the combination study. Median TTP of PSA was 5.8 months. No patients withdrew due to toxicity. No toxic effect was attributed to curcumin. OS was correlated with NE (NSE and CgA) markers.	[175]
NCT03211104	A randomized, double-blind, placebo-controlled trial to evaluate the role of curcumin in prostate cancer patients with intermittent androgen deprivation	NA	Prostate Cancer	Curcumin group: taking curcumin 3 times a day (1440 mg/day) for 6 months from the beginning of androgen deprivation therapy withdrawal. Placebo group: taking placebo for 6 months from the beginning of androgen deprivation therapy withdrawal.	The median off-treatment duration was 16.3 months and 18.5 months in the curcumin group and placebo group, respectively. The proportion of patients with PSA progression during the active curcumin treatment period (6 months) was significantly lower in the curcumin group than the placebo group. Curcumin was well tolerated and safe.	[176]
NCT01917890	Effect of Curcumin Supplementation During Radiotherapy on Oxidative Status of Patients with Prostate Cancer: A Double Blinded, Randomized, Placebo-Controlled Study	NA	Prostate Cancer	Patients received curcumin (3 g) or placebo since 1 week before onset of radiotherapy until completion of their radiotherapy. External beam radiotherapy was given as daily fraction of 2 Gy to achieve a total dose of 74 Gy (5 times a week for about 8 weeks).	Curcumin supplementation did not cause any side effects. In curcumin group, plasma total antioxidant capacity was significantly increased, and the activity of superoxide dismutase decreased after radiotherapy as compared to baseline level and placebo group.	[177]

Note: All completed clinical studies involving curcumin combination therapy found in the literature and ClinicalTrials.gov (accessed on 21 February 2021) are included in the table. BID, twice per day; CEA, carcinoembryonic antigen; CgA, chromogranin A; CRP, C-reactive protein; IL-6, interleukin-6, NA, not available; NE, neuroendocrine; NSE, neuron-specific enolase; ORR, objective response rate; OS, overall survival; PFS, progression free survival; PR, partial response; PSA, prostate-specific antigen; RR, response rate; sCD40L, soluble CD40 ligand; SD, stable disease; TEAE, treatment emergent adverse events; TTP, time to progression; VEGF, vascular endothelial growth factor.

**Table 3:** On-going clinical trial with curcumin combined therapy.

Clinical Trial Number	Title	Phase	Cancer Type	Treatment Regimens	Research Objective
NCT02724202	Curcumin in Combination With 5FU for Colon Cancer	1	Colon cancer	All subjects receive induction oral curcumin 500 mg twice per day for 2 weeks. Patients will continue on curcumin at same dose for an additional 6 weeks while being treated with 3 cycles of 5-FU.	Confirm clinical safety and identify clinical response rate in chemorefractory CRC patients. To determine whether curcumin administration induces systemic alterations in inflammatory and epigenetic biomarkers. To correlate altered biomarker findings with clinical response according to RECIST V1.1 and survival criteria.
NCT02439385	Avastin/FOLFIRI in Combination With Curcumin in Colorectal Cancer Patients With Unresectable Metastasis	2	Colorectal cancer	Patients receive first line Avastin/FOLFIRI in combination with dietary supplementation of nanostructured lipid curcumin particle 100 mg po bid daily.	To evaluate PFS of CRC patients. To evaluate overall survival rate, overall response rate, safety, QoL and fatigue scaling.
NCT01948661	Anthocyanin Extract and Phospholipid Curcumin in Colorectal Adenoma (MIRACOL)	2	Colorectal cancer	Group A: Mirtoselect^®^ 500 mg tablet, 1000 mg (two oral tablets) per day and Meriva^®^ (Curcumin), 500 mg tablet, 1000 mg (two oral tablets) per day for 28 days. Group B: placebo A + placebo B per day for four weeks.	To measure the change of immunohistochemical expression of β-catenin in normal and adenomatous colonic tissue. To measure the change of immunohistochemical expression of NF-κB, Ki-67 Labeling Index and p53 in normal and adenomatous mucosa.
NCT02321293	A Open-label Prospective Cohort Trial of Curcumin Plus Tyrosine Kinase Inhibitors (TKI) for EGFR -Mutant Advanced NSCLC (CURCUMIN)	1	Lung cancer	CurcuVIVA (NPN 80027414) at a single dose of 80 mg PO daily in conjunction with gefitinib 250 mg/erlotinib 150 mg given in a capsule form once daily.	To determine the safety and feasibility of curcumin in chemotherapy. To assess changes in health-related QoL. To evaluate anti-inflammatory properties via CRP measure.
NCT03598309	Phase II Trial to Modulate Intermediate Endpoint Biomarkers in Former Smokers	2	Lung cancer	Group A: 4 g Lovaza^®^, 2 g BID. 8000 mgs CUR Curcumin C3 complex^®^ tablets, 4000 mgs BID. Group B: 2 g Lovaza^®^, 1 g BID. 4000 mgs CUR Curcumin C3 complex^®^ tablets, 2000 mgs BID. Group C: Two matching placebo capsules BID.	To determine the safety and tolerability of treatment. To determine the mean change in bronchial nodule size. To study the rate of adherence per study arm. To evaluate the rate of treatment-related adverse events.
NCT00486460	Phase III Trial of Gemcitabine, Curcumin and Celebrex in Patients With Advance or Inoperable Pancreatic Cancer	3	Pancreatic Cancer	Gemcitabine + Curcumin and/or Celecoxib	To evaluate gemcitabine in combination with curcumin and celecoxib for patients with pancreatic cancer.
NCT04403568	Testing the Synergism of Phytonutrients, Curcumin and Ursolic Acid, to Target Molecular Pathways in the Prostate	1	Prostate Cancer	Cohort 1: Ursolic Acid (150 mg) BID. Cohort 2: Cur (600 mg) BID. Cohort 3: Ursolic Acid (150 mg) + Cur (600 mg) BID. These administrations are subjected to the patients who are scheduled to undergo radical prostatectomy.	To evaluate the bioavailability and safety of treatment. To confirm the presence and levels of ursolic acids and curcumin metabolites in the target organ. To validate the appropriate mechanisms of effect. To determine p65 NF-κB level before and after prostatectomy via immunohistochemistry.
NCT02724618	Nanocurcumin for Prostate Cancer Patients Undergoing Radiotherapy (RT)	2	Prostate Cancer	Experimental group: 120 mg/d oral nanocurcumin (3 capsules of SinaCurcumin^®^ 40 per day) 3 days before and during radiotherapy + external beam radiation therapy Placebo comparator: placebo (3 placebo capsules of SinaCurcumin^®^ 40 per day), 3 days before and during radiotherapy + external beam radiation therapy.	To determine the cystitis, hematologic toxicity, biochemical PFS and treatment response.
NCT04731844	Curcumin and Piperine in Patients on Surveillance for Monoclonal Gammopathy, Smoldering Myeloma or Prostate Cancer	2	Prostate Cancer, Multiple Myeloma, Smoldering Multiple Myeloma, Monoclonal Gammopathy of Undetermined Significance	Experimental group: curcumin plus piperine at a dose of 4 g/5 mg orally BID for 12 months.	To determine the response rate of curcumin and piperine supplementation in patients. To determine the PFS.

Note: All on-going clinical studies involving curcumin combination therapy found in the literature and ClinicalTrials.gov (accessed on 21 February 2021) are included in the table. BID, twice a day CRC, colorectal cancer; CRP, C-reactive protein; PFS, progression free survival; QoL, quality of life.
